# Pain Management Strategies among Cancer Patients. Normalization of the CSQ (The Pain Coping Strategies Questionnaire) Form

**DOI:** 10.3390/ijerph19021013

**Published:** 2022-01-17

**Authors:** Urszula Religioni, Aleksandra Czerw, Katarzyna Sygit, Krzysztof Zdziarski, Olga Partyka, Monika Pajewska, Anna Augustynowicz, Michał Waszkiewicz, Elżbieta Cipora, Ewa Ziółkowska, Dominika Mękal, Sylwia Jopek, Łukasz Strzępek, Tomasz Banaś

**Affiliations:** 1 Collegium of Business Administration, Warsaw School of Economics, 02-513 Warsaw, Poland; urszula.religioni@gmail.com; 2 School of Public Health, Centre of Postgraduate Medical Education of Warsaw, 01-813 Warsaw, Poland; aaugustynowicz@poczta.onet.pl (A.A.); michal.waszkiewicz@cmkp.edu.pl (M.W.); 3 Department of Health Economics and Medical Law, Medical University of Warsaw, 02-091 Warsaw, Poland; sylwia.jopek@wum.edu.pl; 4 Department of Economic and System Analyses, National Institute of Public Health NIH-National Research Institute, 00-791 Warsaw, Poland; opartyka@pzh.gov.pl (O.P.); mpajewska@pzh.gov.pl (M.P.); 5 Faculty of Health Sciences, Calisia University, 62-800 Kalisz, Poland; ksygit@poczta.onet.pl; 6 Subdepartment of Social Medicine and Public Health, Department of Social Medicine, Pomeranian Medical University in Szczecin, 70-204 Szczecin, Poland; krzysztof.zdziarski@pum.edu.pl; 7 Faculty of Medical Science, Jan Grodek State University in Sanok, 38-500 Sanok, Poland; elacipora@interia.pl; 8 Interfaculty Department of Mechanical and Medical Engineering, Calisia University, 62-800 Kalisz, Poland; e.ziolkowska@poczta.onet.pl; 9 Department of Cancer Prevention, Medical University of Warsaw, 02-091 Warsaw, Poland; dmekal@wum.edu.pl; 10 Department of General Surgery, Regional Public Hospital in Bochnia, 32-700 Bochnia, Poland; strzepeklukasz@wp.pl; 11 Department of Gynecology and Obstetrics, Jagiellonian University Medical College, 31-501 Krakow, Poland; tbanas@mp.pl

**Keywords:** cancer patients, cancer, CSQ, normalization

## Abstract

Introduction: Cancer is associated with chronic pain, which significantly reduces the quality of life. The level of pain depends on the dominant pain management strategy that the patient uses. Objective: This study seeks to evaluate the application of the Pain Coping Strategies Questionnaire among cancer patients and develop norms allowing differentiation of patients with diagnosed cancer in terms of pain management strategies. Material and Methods: The study involved 1187 patients diagnosed with malignant cancer, who are under outpatient care of the Maria Sklodowska-Curie Institute—Oncology Center in Warsaw. The study used the Pain Coping Strategies Questionnaire (CSQ) elaborated by A.K. Rosentel and F.J. Keefe. Results: Socioeconomic variables and medical factors affect pain management strategies chosen by patients. The area most strongly differentiated by the studied variables is praying/hoping. Factors that have the greatest impact on the choice of pain management strategies for cancer patients include education, income, and radiation therapy. Sten standards were developed to determine the severity of pain management strategies used in the low-average-high categories. Conclusions: The CSQ questionnaire should be used in cancer patients, and the result of the strategy used can be a prognostic factor for the expected effects of therapy. Knowledge of the variables most strongly affecting patients’ choice of strategies that are not conducive to strengthening health attitudes and the ability to determine the severity of pain management strategies on standard scales allows us to focus psychotherapeutic activities on patients who need support most.

## 1. Introduction

Cancer is the second most common cause of death for Poles (right after cardiovascular diseases), causing 27.3% of deaths among men and almost 24.1% of deaths among women in 2016. The most common cancer in Poland in the female population include breast cancer, lung cancer, and endometrial cancer, and in the male population, prostate cancer, lung cancer, and colorectal cancer. The dominant causes of cancer-related deaths in women include lung cancer, breast cancer, and endometrial cancer, and in men, lung cancer, prostate cancer, and colorectal cancer [1].

Cancer causes a significant reduction in the quality of life of patients, mainly through frequent pain, which is defined as an unpleasant sensory and emotional experience associated with existing or possible tissue damage [2]. Pain is subjective and related to the patient’s previous experience of pain, knowledge of its causes, treatment or consequences of pain, as well as the degree of emotional arousal [3]. It is indicated that pain may also be physiological (warning about a threat) or pathological in situations where it accompanies the disease [2].

Pain and high levels of stress are some of the most common conditions occurring in the case of cancer. By influencing each other, they reinforce each other’s subjective impact and reduce the patient’s quality of life [4].

The feeling of pain and approach to stressful situations, and thus the quality of life, is largely influenced by how the patient copes with the disease. Coping means that the patient is making efforts to master external and internal requirements that the person assesses as burdening or exceeding his/her resources. Coping can focus on a task (when the patient with chronic pain is focused on maintaining activity and pain-reducing actions), emotions (when it is focused on controlling the emotional response associated with the given stressor) and on avoiding and undertaking substitute activities. The situation and the way it is interpreted and assessed by the patient have the greatest impact on the choice of coping strategies [2].

One of the questionnaires measuring how to deal with pain is the Pain Coping Strategies Questionnaire (CSQ), elaborated by A.K. Rosentel and F.J. Keefe [5]. The CSQ questionnaire has been translated and adapted in many countries, including Germany [6]. It serves to assess strategies used in relation to experienced pain, as well as their effectiveness in pain management, which is applicable in both diagnosis and therapy of patients complaining of pain [5]. Although the results of normalization are not widely published in the literature, we believe that this procedure is necessary to establish the norms of the results of the questionnaire for specific groups of patients, in this case, oncological patients. In the process of literature review, sources of medical information were searched. Scientific articles present in PubMed, Scopus, Cohrane Library were included. The following key words present in databases were included in the recognition/research strategy in different combinations: CSQ questionnaire; Pain Coping Strategies Questionnaire; normalization; cancer.

The objective of the study is to normalize the Pain Coping Strategies Questionnaire (CSQ) in a group of cancer patients. Normalization allows to establish norms of test results for a specific population. This procedure allows to understand what the result obtained by a specific person in the questionnaire means, including whether the result is high or low.

## 2. Materials and Methods

### 2.1. Characteristics of the Studied Normalization Group

The criteria for enrolling patients was the patient’s availability at the Maria Sklodowska-Curie Institute—Oncology Center in Warsaw during conducting the study and the patient’s consent to participate in the study.

The study involved 1187 patients (666 women and 521 men) who were diagnosed with cancer. The most common cancers in the group of women were breast cancer (29% of the studied group of women), ovarian cancer (25.8%) and endometrial cancer (17.4%), and in the group of men, prostate cancer (43.8% of the group of men), colorectal cancer (26.9%), and bladder cancer (15.9%). Table 1 presents a detailed structure of the sample, including the location of cancer.

Respondents were 21–96 years old (M = 62.12; SD = 14.03); the age range for women was 21–96 years (M = 58.17; SD = 12.88), and for men 22–96 years (M = 67.12; SD = 13.75). In the studied group, 8.3% of patients had primary education, 21.1% vocational, 37.7% secondary and 33.1% higher education. The largest group lived in cities over 500,000 residents (36%) and in the countryside (18.6%). Others lived in small towns up to 500,000 residents.

The average monthly income per person in a household was as follows: up to PLN 500—2%, PLN 501–1000—17.7%, PLN 1001–1500—26.7%, PLN 1501–2000—25.2%, above PLN 2000—27.7%. The usual currency exchange rate is more than 4.5 PLN for 1 euro. Of the studied group of patients, 52.1% were pensioners, 39.1% were working people, 4.5% were homeowners, 2.8% were unemployed, and 1.6% were students. The largest group of people was married (68.4%), in the group there were 16.2% of widows/widowers, 7.8% were single and 7.6% divorced. Table 2 summarizes age distribution and indicators of the advancement of the neoplastic disease.

The age of most respondents was within the range from 40 to 65 years. Most patients were undergoing chemotherapeutic treatment. Fewer patients were undergoing radiotherapy and targeted treatment.

### 2.2. Applied Tool

The Pain Coping Strategies Questionnaire (CSQ) is intended to study patients suffering from pain. The questionnaire consists of 42 statements describing different types of pain management and two questions regarding the assessment of one’s own coping and pain reduction skills. In each of the questions, the patient uses the Likert scale to assess the frequency of behavior in a given way or the degree of pain management. A score from 0–36 points is calculated for each strategy. The higher the score, the greater the patient’s level of pain management.

The ways of coping with pain reflect six cognitive strategies (distraction, re-evaluation of pain, catastrophizing, ignoring sensations, praying/hoping, declaring coping) and one behavioral strategy (increased behavioral activity), which are part of three factors: cognitive coping (strategies for re-evaluating pain, ignoring sensations and declaring coping), distraction and taking substitute activities (distraction, increased behavioral activity), and catastrophizing and seeking hope (catastrophizing and praying/hoping).

Internal compliance of the CSQ Pain Coping Strategies Questionnaire was assessed on the basis of a study of patients with chronic pain. Cronbach’s alpha for the entire questionnaire is 0.80; for five scales the coefficients exceed 0.80, for two (distraction and increased behavioral activity) they are lower and are 0.64 and 0.63, respectively. These indicators are similar to those obtained in the original version of the questionnaire [5].

### 2.3. Statistics

Statistical analysis was based on between group comparisons. Depending on CSQ scores, distribution parametric on non-parametric statistical tests were used. For comparisons between two groups either parametric Student’s *t*-test or non-parametric Mann–Whitney test was used. For comparisons between three groups either parametric one-way analysis of variance or Kruskal–Wallis test was used.

## 3. Results

### 3.1. Descriptive Statistics

Analyzing the impact of medical variables on pain management strategies chosen by patients, the results indicate that distraction is differentiated by chemotherapeutic treatment (*p* = 0.009) and radiation therapy (*p* = 0.040), with higher results in this area characterizing patients undergoing these types of treatment.

Re-evaluation of pain is differentiated by radiation therapy (*p* = 0.012). Patients who have undergone radiation therapy attribute greater importance to this strategy.

The average result of catastrophizing is influenced by the fact that patients have metastases (*p* = 0.001), they are undergoing chemotherapeutic treatment (*p* = 0.010) and targeted treatment (*p* = 0.008). The occurrence of metastases or chemotherapy or targeted therapy contributed to the intensification of catastrophizing.

Ignoring pain was differentiated by radiation therapy (*p* = 0.040), and patients undergoing radiation therapy obtained a higher result in this area.

Praying/hoping depended on the occurrence of metastases (*p* = 0.007), chemotherapeutic treatment (*p* = 0.005) and radiation therapy (*p* = 0.035). The occurrence of metastases or chemotherapy or radiation therapy resulted in higher results for praying/hoping.

No influence of medical variables on coping declarations was observed in the studied patients. Increased behavioral activity was differentiated by radiation therapy (*p* = 0.029), and patients undergoing this type of treatment were characterized by intensification of this strategy. Table 3 presents detailed relationships.

Analysis of socioeconomic variables affecting pain management strategies in cancer patients showed the impact of these variables on all areas of pain management, and detailed results are presented in Table 4.

Distraction is differentiated by education (*p* = 0.001) and patient income (*p* = 0.008). The higher the patient’s education and the higher the income, the smaller the significance of this strategy in fighting pain.

Re-evaluation of pain is affected by education (*p* = 0.004) and income (*p* = 0.021). This strategy is most important in patients with lower education and lower income.

Catastrophizing is differentiated by patients’ gender (*p* = 0.001), where women tend to catastrophize more than men, using it as a pain management strategy.

Ignoring pain, in turn, depends on patients’ education (*p* = 0.003) and is most important in patients with primary/vocational education, and the least in patients with higher education.

Praying/hoping is differentiated by gender (*p* = 0.001), education (p = 0.001), place of residence (*p* = 0.014) and income (*p* = 0.001). The strategy of praying/hoping is more important for women than men, for people with primary/vocational education than secondary and higher education, for people from smaller towns and with lower incomes.

The average intensity of coping strategies depends on the patient’s place of residence (*p* = 0.048) and is greater for those living in larger cities (>1,000,000 residents).

Increased behavioral activity is differentiated by education (*p* = 0.009), and its average score increases as the education of the respondents decreases.

### 3.2. Norms

Table 5 and Table 6 present the values of sten and centile norms determined using the calculated weight for individual areas of the CSQ test. Due to statistically significant differences between the genders in terms of results on the scale of catastrophizing and praying/hoping, norms for these two dimensions were developed separately for women and men. The remaining scales were normalized throughout the entire sample. These tables make it possible to transform raw results of an individual into normalized results. The normalized results can then be interpreted as low, average, or high in relation to results in the population of patients diagnosed with cancer.

## 4. Discussion

Pain management strategies are actions taken by patients in situations of pain. Their effectiveness may depend on the level of pain and stress experienced in the face of the disease, as well as on the individual characteristics of the patient [3]. The study of cancer patients indicates that they most often apply strategies, such as declaring coping, praying/hoping, increased behavioral activity or distraction. Our results show that individual dimensions of pain management strategies are strongly differentiated by the level of education, income, or the use of radiation therapy. This information is a valuable source, especially for clinical psychologists, who can more easily identify the groups of people who should receive psychological support, especially in the area of increasing self-confidence in the fight against the disease.

The study by Ślusarska, B. et al., including patients with various types of cancer receiving hospice care, indicates that they cope with pain most by praying and hoping (M = 26.92), declaring coping (M = 17.65) and catastrophizing (M = 14.85), and these results are differentiated by education (people with higher education more often used the strategy of increased behavioral activity), place of residence (rural residents more often chose the strategy of praying/hoping, increased behavioral activity and declaration of coping, and less often catastrophizing) [4].

While comparing strategies for coping with pain among cancer patients and healthy people, Ahadi H. et al. stated that cancer patients mainly use strategies to cope with emotions and avoidance and are less likely to take responsibility for pain [7].

When studying patients with bladder cancer, Krajewski W. et al. indicated that coping strategies (M = 18.37) and praying/hoping (M = 17.7) dominated among them [8].

In the case of patients with non-cancerous diseases, patients adopt similar pain management strategies. For example, patients with degenerative changes of the hip joint use praying/hoping (M = 23.30) and declaring coping (20.99) [9] to the greatest extent, which is also confirmed by Andruszkiewicz A. [3]. Similarly, praying/hoping and declaring coping are the most commonly chosen strategies for fighting pain among women with coronary heart disease [10].

Patients with internal medicine diseases are also mostly characterized by praying/hoping followed by increased behavioral activity and declaring coping. In this case, a significant relationship was observed between the choice of strategy and the severity of pain, education and professional activity of patients [11].

Among patients with chronic neuropathic pain, the most commonly chosen strategies were praying/hoping (M = 17.72) and declaring coping (M = 17.39) [12]. These strategies also dominated in the study of patients with rheumatoid arthritis, reaching M = 22.4 for praying/hoping and M = 21.0 for declaring coping [13]. Although the study conducted by Kwissy-Gajewska Z. et al. confirms that patients with RA (rheumatoid arthritis) mostly apply the strategy of praying/hoping [14], another study of patients with rheumatoid arthritis indicates that the dominant strategies in these patients are increased behavioral activity and declaring coping [15].

Patients with lower limb ischemia experiencing chronic pain are characterized by catastrophizing and praying/hoping strategies [16]. Among patients qualified for surgery due to osteoarthritis of the spine, strategies such as praying/hoping and declaring coping dominate [17].

Praying/hoping and declaring coping are also the main pain management strategies among women with endometriosis (the average values for these areas are M = 20.8 and M = 19.00, respectively). Women with endometriosis rarely use strategies to ignore pain, re-evaluate pain, and catastrophize [18].

In literature, gender differences are particularly emphasized, in which it is indicated that men think that they can control their pain to a greater extent, and more often they deny the feeling of pain and are engaged with substitute activities, diverting attention from the pain they feel. Women, in turn, are more focused on pain. They are more often looking for social support, but they have a tendency to catastrophize [2,19].

Studies indicate that patients assessing their quality of life higher more often use coping strategies such as distraction, re-evaluation of pain, ignoring pain, declaring coping, and increased behavioral activity [20], although the impact of pain coping strategies on the acceptance of disease is not shown [17].

Therefore, the choice of pain management strategies significantly affects the patient’s functioning. Catastrophizing is especially widely described in literature in this context, which according to many authors, is associated with stronger pain experienced by the patient, greater disability, or worse mental condition [21,22].

Similar relationships were observed in the area of praying/hoping. People who use this strategy more often feel hopelessness, tend to catastrophize, feel pain more and have a higher degree of disability [5], although some studies do not confirm these relationships [23].

Similarly, results of studies conducted by various authors differ in relation to the impact of strategies of increased behavioral activity and ignoring pain on the patient’s pain and functioning [16,24,25].

## 5. Conclusions

The Pain Coping Strategies Questionnaire (CSQ) is applicable to cancer patients. Considering the results of studies showing that attitudes of coping with pain affect the effectiveness of treatment, pain or disability progression, the assessment of pain management by cancer patients may be a prognostic factor regarding the effects of therapy. Knowledge of demographic and medical aspects affecting patients’ choice of pain management strategies and the ability to determine the results in an individual CSQ questionnaire on normalized scales can constitute the basis for planning psychotherapeutic actions aimed at those patients who need to modify their behavior and attitudes towards pain the most to avoid the approach based on catastrophizing and praying/hoping. Changing the strategy of coping with pain by patients will reduce the incidence of anxiety, depression, and disability, and it will improve the patient’s prognosis.

### Limitations

Our study has limitations, which include, first of all, the lack of checking the influence of the stage of the disease, the specific type of treatment, ability to perform activities of daily living, use of opiates, or psychiatric medicine and/or illicit drugs/alcohol on the results obtained. Further research in this area may reveal additional links between pain control and other factors that affect the type of pain control in a patient.

## Figures and Tables

**Table 1:** Structure of the studied sample, including cancer location in relation to the population.

	Women			Men		
Cancer Location	Population(%)	Sample(%)	Weight	Population(%)	Sample(%)	Weight
breast cancer	21.9	29.0	0.76	0	0	-
ovarian cancer	4.7	25.8	0.18	0	0	-
stomach cancer	2.4	6.8	0.35	4.5	9.2	0.49
colorectal cancer	10.1	14.9	0.68	12.2	26.9	0.45
prostate cancer	0	0	-	15.5	43.8	0.35
bladder cancer	2.0	2.6	0.77	6.9	15.9	0.43
endometrial cancer	7.3	17.4	0.42	0	0	-
pancreatic cancer	2.2	3.6	0.61	2.3	4.2	0.55

**Table 2:** Age distribution and indicators of the advancement of the neoplastic disease.

Variables		n	%
Age	20–40	94	7.9
	40–65	574	48.4
	65+	516	43.5
	missing data	3	0.3
Metastases		349	29.4
Chemotherapeutic treatment		444	37.4
Radiotherapy		154	13.0
Targeted treatment		89	7.5

**Table 3:** Disease indicators differentiating CSQ test results.

Variables		M	SD	Min	Max	Test	*p*
Distraction							
Chemotherapy	yes	20.33	8.46	0	36	t(550) = 2.64	0.009
	no	18.24	9.24	0	36		
Radiation therapy	yes	21.25	8.11	0	36	t(550) = 2.29	0.040
	no	18.67	9.10	0	36		
Re-evaluation of pain							
Radiation therapy	yes	14.17	8.43	0	31	U = 6164.50	0.012
	no	11.87	9.29	0	36		
Catastrophizing							
Metastases	yes	12.38	8.53	0	36	U = 9666.50	0.001
	no	10.01	8.02	0	36		
Chemotherapy	yes	12.09	8.38	0	36	U = 12,080.00	0.010
	no	10.13	8.05	0	36		
Targeted	yes	14.23	7.40	0	29	U = 3187.00	0.008
therapy	no	10.57	8.23	0	36		
Ignoring pain							
Radiation therapy	yes	17.68	8.72	0	36	t(550) = 2.06	0.040
	no	15.24	9.53	0	36		
Praying/hoping							
Metastases	yes	21.49	9.16	0	36	t(516) = 2.71	0.007
	no	19.01	9.85	0	36		
Chemotherapy	yes	21.43	9.12	0	36	t(550) = 2.84	0.005
	no	19.00	9.93	0	36		
Radiation therapy	yes	22.11	8.26	0	36	t(550) = 2.11	0.035
	no	19.55	9.87	0	36		
Increased behavioral activity							
Radiation therapy	yes	22.34	8.06	0	36	U = 6382.50	0.029
	no	20.20	9.31	0	36		

M—median value; SD—standard deviation; min—minimum value; max—maximum value; t—Student’s *t*-test value for independent samples; U—U Mann–Whitney test value; df—number of degrees of freedom; *p*—statistical significance.

**Table 4:** Socioeconomic variables that differentiate CSQ test results.

Variables		M	SD	Min	Max	Test	*p*
Distraction							
Education	prim./voc.	20.59	8.37	0	36	*F*(2.549) = 6.94	0.001
	secondary	19.46	8.94	0	36		
	higher	17.07	9.35	0	36		
Net	up to PLN 1500 (46.4%)	20.05	8.57	0	36	*t*(550) = 2.65	0.008
income	above PLN 1500 (52.9%)	18.03	9.32	0	36		
Re-evaluation of pain							
Education	prim./voc.	13.74	9.29	0	36	χ^2^(2) = 11.07	0.004
	secondary	12.22	9.32	0	36		
	higher	10.74	8.81	0	36		
Net	Up to PLN 1500 (46.4%)	12.90	9.24	0	35	U = 13,454.50	0.021
income	above PLN 1500 (52.9%)	11.49	9.14	0	36		
Catastrophizing							
Gender	women	11.56	8.17	0	36	*U* = 1844.00	0.001
	men	9.74	8.18	0	36		
Ignoring pain							
Education	prim./voc.	17.10	9.63	0	36	*F*(2.549) = 5.89	0.003
	secondary	15.98	9.21	0	36		
	higher	13.69	9.32	0	36		
Praying/hoping							
Gender	women	21.29	9.17	0	36	*t*(424.73) = 4.20	0.001
	men	17.71	10.13	0	36		
Education	prim./voc.	21.83	9.83	0	36	*F*(2.549) = 10.85	0.001
	secondary	20.67	9.52	0	36		
	higher	17.24	9.29	0	36		
Size of	up to 100,000 residents (54.7%)	20.82	9.68	0	36	*t*(550) = 2.75	0.014
Place of residence	above 100,000 residents (45.3%)	18.77	9.63	0	36		
Net	up to PLN 1500	21.96	9.45	0	36	*t*(550) = 4.96	0.001
income	above PLN 1500 (52.9%)	17.95	9.55	0	36		
Declaring coping							
Size of	up to 100,000 residents (54.7%)	20.66	9.36	0	36	*U* = 13,685.50	0.048
Place of residence	above 100,000 residents (45.3%)	21.07	9.88	0	36		
Increased behavioral activity							
Education	prim./voc.	21.58	8.71	0	36	χ^2^(2) = 9.48	0.009
	secondary	20.84	9.04	0	36		
	higher	19.08	9.59	0	36		

M—median value; SD—standard deviation; min—minimum value; max—maximum value; t—Student’s *t*-test value for independent samples; F—one-way analysis of variance value; U—U Mann–Whitney test value; χ2–H Kruskal–Wallis test value; df—number of degrees of freedom; *p*—statistical significance.

**Table 5:** Raw results and corresponding normalized results for distraction, re-evaluation of pain, ignoring pain, declaring coping, and increased behavioral activity in the entire sample studied.

Distraction				Re-Evaluation of Pain				Ignoring Pain				Declaring Coping				Increased Behavioral Activity			
Results	Value	Sten	Centile	Results	Value	Sten	Centile	Results	Value	Sten	Centile	Results	Value	Sten	Centile	Results	Value	Sten	Centile
low	0	2	3	low	0	3	8	low	0	2	4	low	0	2	3	low	0	2	2
	1	2	6	average	1	4	16	average	1	3	9		1	2	6		1	2	5
	2	2	6		2	4	18		2	3	10		2	2	6		2	2	6
	3	3	7		3	4	22		3	3	12		3	3	7		3	2	6
	4	3	8		4	4	26		4	3	15		4	3	8		4	3	7
	5	3	9		5	4	28		5	4	16		5	3	8		5	3	7
	6	3	11		6	5	32		6	4	19		6	3	9		6	3	8
	7	3	13		7	5	35		7	4	22		7	3	10		7	3	10
	8	3	14		8	5	37		8	4	23		8	3	11		8	3	10
average	9	4	16		9	5	41		9	4	26		9	3	13		9	3	12
	10	4	18		10	5	45		10	4	29		10	3	14		10	3	14
	11	4	19		11	5	48		11	5	32		11	3	15	average	11	4	16
	12	4	22		12	6	51		12	5	35	average	12	4	17		12	4	18
	13	4	24		13	6	55		13	5	39		13	4	20		13	4	21
	14	4	26		14	6	58		14	5	42		14	4	22		14	4	23
	15	4	29		15	6	61		15	5	46		15	4	25		15	4	26
	16	5	33		16	6	64		16	6	51		16	4	28		16	4	29
	17	5	37		17	6	67		17	6	55		17	5	31		17	5	31
	18	5	41		18	7	71		18	6	61		18	5	35		18	5	36
	19	5	46		19	7	76		19	6	66		19	5	40		19	5	40
	20	5	50		20	7	80		20	7	69		20	5	43		20	5	45
	21	6	54		21	7	82		21	7	72		21	5	47		21	5	49
	22	6	58	high	22	8	85		22	7	75		22	6	51		22	6	53
	23	6	62		23	8	86		23	7	77		23	6	55		23	6	57
	24	6	68		24	8	88		24	7	80		24	6	60		24	6	61
	25	7	74		25	8	90		25	7	83		25	6	64		25	6	66
	26	7	77		26	8	92	high	26	8	85		26	6	68		26	6	69
	27	7	81		27	8	93		27	8	88		27	7	71		27	7	72
high	28	8	85		28	9	94		28	8	89		28	7	74		28	7	76
	29	8	87		29	9	95		29	8	91		29	7	77		29	7	79
	30	8	90		30	9	97		30	8	93		30	7	81	high	30	8	84
	31	8	92		31	10	98		31	9	94	high	31	8	85		31	8	89
	32	9	94		32	10	98		32	9	95		32	8	87		32	8	91
	33	9	96		33	10	99		33	9	96		33	8	90		33	9	93
	34	9	97		34	10	99		34	9	97		34	8	92		34	9	95
	35	10	98		35	10	100		35	9	97		35	9	94		35	9	97
	36	10	99		36	10	100		36	10	99		36	9	97		36	10	99

**Table 6:** Raw results and corresponding normalized results for catastrophizing and praying/hoping in groups of men and women.

Catastrophizing								Praying/Hoping							
Women				Men				Women				Men			
Results	Value	Sten	Centile	Results	Value	Sten	Centile	Results	Value	Sten	Centile	Results	Value	Sten	Centile
low	0	2	6	low	0	3	10	low	0	2	2	low	0	2	5
	1	3	14	average	1	4	22		1	3	4		1	3	10
	2	3	16		2	4	24		2	3	4		2	3	11
average	3	4	18		3	4	27		3	3	5		3	3	12
	4	4	22		4	5	31		4	3	6		4	3	13
	5	4	25		5	5	35	average	5	4	6		5	3	14
	6	4	29		6	5	40		6	4	7		6	3	16
	7	5	34		7	5	44		7	4	9	average	7	4	18
	8	5	37		8	5	48		8	4	10		8	4	19
	9	5	41		9	6	51		9	4	11		9	4	21
	10	5	46		10	6	54		10	4	13		10	4	23
	11	5	50		11	6	58		11	5	14		11	4	26
	12	6	52		12	6	61		12	5	16		12	4	29
	13	6	57		13	6	65		13	5	18		13	5	32
	14	6	61		14	7	69		14	5	20		14	5	34
	15	6	65		15	7	73		15	5	22		15	5	38
	16	7	70		16	7	76		16	6	25		16	5	40
	17	7	74		17	7	78		17	6	28		17	5	44
	18	7	77		18	7	82		18	6	33		18	5	48
	19	7	80		19	8	85		19	6	38		19	6	52
	20	7	84		20	8	88		20	7	41		20	6	56
high	21	8	87	high	21	8	90		21	7	46		21	6	61
	22	8	90		22	8	92		22	7	51		22	6	65
	23	8	91		23	9	94		23	7	55		23	6	68
	24	8	93		24	9	95		24	7	60		24	7	72
	25	9	95		25	9	96		25	7	64		25	7	75
	26	9	96		26	9	97	high	26	8	67		26	7	78
	27	9	97		27	9	97		27	8	70		27	7	81
	28	9	98		28	9	98		28	8	75		28	7	83
	29	10	98		29	10	98		29	8	78	high	29	8	85
	30	10	98		30	10	98		30	8	82		30	8	88
	32	10	99		32	10	99		31	9	86		31	8	90
	33	10	99		33	10	99		32	9	88		32	8	91
	34	10	99		34	10	99		33	9	89		33	8	92
	35	10	99		35	10	99		34	9	92		34	9	94
	36	10	100		36	10	100		35	9	93		35	9	95
									36	10	97		36	10	98

## Data Availability

The data analyzed during the current study are available at authors.
